# P-1506. Improving Meningococcal Vaccine Awareness and Engagement in Boarding School and College Residence Halls

**DOI:** 10.1093/ofid/ofaf695.1690

**Published:** 2026-01-11

**Authors:** Shaina Y Vincent, Kelly E Pillinger, Leah Molloy, Laura Simone, Chris Napolitan, Jeffrey D Carter, Melissa Rodriguez, Jenniffer A Meza Jimenez, Gary S Marshall

**Affiliations:** PRIME Education, Shelby Twp, Michigan; PRIME Education, Shelby Twp, Michigan; PRIME Education, Shelby Twp, Michigan; PRIME Education, LLC, Fort Lauderdale, Florida; PRIME Education, Shelby Twp, Michigan; PRIME Education, LLC, Fort Lauderdale, Florida; PRIME Education, Shelby Twp, Michigan; PRIME Education, Shelby Twp, Michigan; Norton Children's and University of Louisville School of Medicine, Louisville, KY

## Abstract

**Background:**

Adolescents and young adults living in residence halls are at increased risk of invasive meningococcal disease (IMD), but vaccination rates are suboptimal. This project aimed to raise awareness of IMD and vaccination among students in residential educational environments.

**Table 1.**
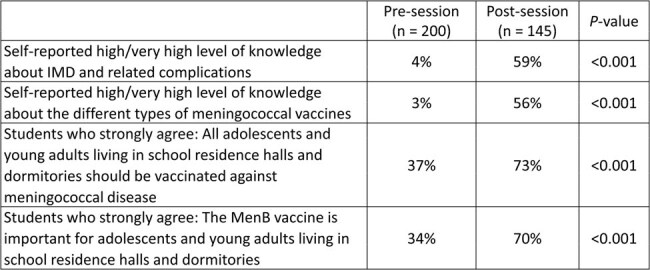
Knowledge and Attitudes Regarding Invasive Meningococcal Disease (IMD) and Vaccination Among Student Attendees

**Figure 1.**
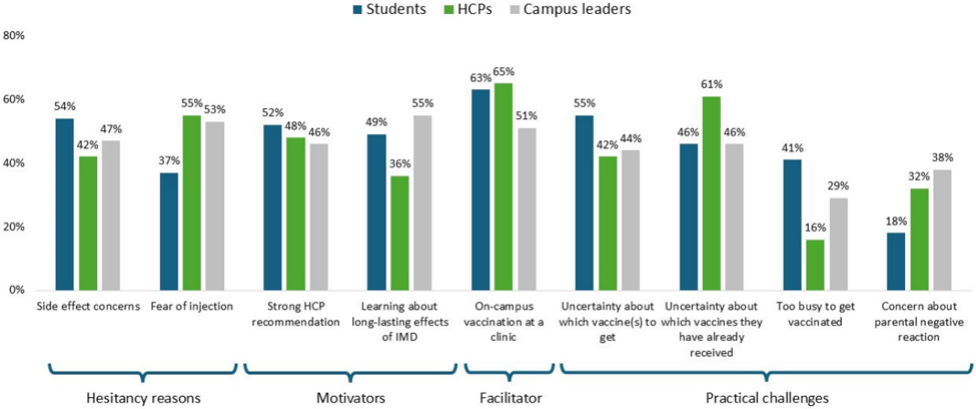
Perceptions Regarding Student Meningococcal Vaccination Acceptance

**Methods:**

In 2024, collaborative learning sessions (CLS) were held for students at 1 boarding high school and 4 residential colleges in 5 states (AL, CA, DC, FL, and TX). Trained local healthcare professionals (HCPs) led the CLS, and campus leaders also attended to facilitate subsequent discussions with students. Tethered surveys were given to students, HCPs, and campus leaders before and after the CLS and 4-6 weeks later. Content was shared nationally through an online toolkit and on-demand video.

**Results:**

Overall, 200 students (median age 20 years; 66% female; 7% unvaccinated; 40% unsure of vaccination status), 31 HCPs, and 55 campus leaders participated in the CLS. Self-reported knowledge and attitudes regarding IMD and vaccination significantly improved (Table). 99% of unvaccinated students reported being likely to get vaccinated post-CLS. Shared and discordant perceptions regarding student vaccine acceptance were observed (Figure). Students’ top reason for vaccine hesitancy was potential side effects, while HCPs/campus leaders believed fear of injection caused their hesitancy. Across all groups, strong HCP recommendations, learning about IMD, and on-campus vaccination were key motivators and facilitators, while unknown vaccine history and confusion about what vaccines to get were agreed upon as major barriers. In addition, students cited being too busy as a barrier, unlike HCPs/campus leaders who anticipated negative parental reactions as a major concern. 4-6 weeks later, 93 students (107 did not complete the survey) reported their vaccination status: 64 (69%) reported receiving MenACWY and 52 (56%) reported receiving MenB vaccine since the CLS. As of February 2025, the toolkit and video were accessed by 1,162 and 1,337 HCPs, respectively.

**Conclusion:**

Targeted education of residential students and campus leaders improved meningococcal immunization rates and revealed perceptions about student vaccination acceptance.

**Disclosures:**

Gary S. Marshall, MD, GSK: Advisor/Consultant|GSK: Grant/Research Support|Merck: Advisor/Consultant|Merck: Grant/Research Support|Moderna: Advisor/Consultant|Pfizer: Advisor/Consultant|Pfizer: Grant/Research Support|Sanofi: Advisor/Consultant|Sanofi: Grant/Research Support|Seqirus: Advisor/Consultant

